# Comparison of telemonitoring combined with intensive patient support with standard care in patients with chronic cardiovascular disease - a randomized clinical trial

**DOI:** 10.1186/s40001-023-00991-1

**Published:** 2023-01-11

**Authors:** Alper Öner, Hermann Dittrich, Fatih Arslan, Sissy Hintz, Jasmin Ortak, Bernard Brandewiede, Miriam Mann, Katja Krockenberger, Alexandre Thiéry, Andreas Ziegler, Christian Schmidt, the CardioCare MV Study Group, H Bleschke, T Buchner, C Buckow, K Bunge, S Duda, H El-Sourani, K Frey, H Greiner-Leben, F Henschel, R Hering, O Knispel, J Kram, A Martschewski, R Mitusch, S Plietzsch, S Rausch, A Rink, M Wejda, R Wißmann, B Wolf.

**Affiliations:** 1 Department of Cardiology, Zentrum Für Innere Medizin (ZIM), Universitätsmedizin Rostock, Ernst-Heydemann-Str. 6, 18057 Rostock, Germany; 2 Department of Cardiology, Leiden University Medical Center, Leiden, The Netherlands; 3 AMEDON GmbH, Lübeck, Germany; 4 Cardio-CARE, Medizincampus Davos, Davos, Switzerland; 5 School of Mathematics, Statistics and Computer Science, University of KwaZulu-Natal, Pietermaritzburg, South Africa; 6 Department of Cardiology, University Heart and Vascular Center Hamburg, Hamburg, Germany

## Abstract

**Importance:**

Healthcare concepts for chronic diseases based on tele-monitoring have become increasingly important during COVID-19 pandemic.

**Objective:**

To study the effectiveness of a novel integrated care concept (NICC) that combines tele-monitoring with the support of a call centre in addition to guideline therapy for patients with atrial fibrillation, heart failure, or treatment-resistant hypertension.

**Design:**

A prospective, parallel-group, open-label, randomized, controlled trial.

**Setting:**

Between December 2017 and August 2019 at the Rostock University Medical Center (Germany).

**Participants:**

Including 960 patients with either atrial fibrillation, heart failure, or treatment-resistant hypertension.

**Interventions:**

Patients were randomized to either NICC (*n* = 478) or standard-of-care (SoC) (*n* = 482) in a 1:1 ratio. Patients in the NICC group received a combination of tele-monitoring and intensive follow-up and care through a call centre.

**Main outcomes and measures:**

Three primary endpoints were formulated: (1) composite of all-cause mortality, stroke, and myocardial infarction; (2) number of inpatient days; (3) the first plus cardiac decompensation, all measured at 12-months follow-up. Superiority was evaluated using a hierarchical multiple testing strategy for the 3 primary endpoints, where the first step is to test the second primary endpoint (hospitalization) at two-sided 5%-significance level. In case of a non-significant difference between the groups for the rate of hospitalization, the superiority of NICC over SoC is not shown.

**Results:**

The first primary endpoint occurred in 1.5% of NICC and 5.2% of SoC patients (OR: 3.3 [95%CI 1.4–8.3], *p* = 0.009). The number of inpatient treatment days did not differ significantly between both groups (*p* = 0.122). The third primary endpoint occurred in 3.6% of NICC and 8.1% of SoC patients (OR: 2.2 [95%CI 1.2–4.2], *p* = 0.016). Four patients died of all-cause death in the NICC and 23 in the SoC groups (OR: 4.4 [95%CI 1.6–12.6], *p* = 0.006). Based on the prespecified hierarchical statistical analysis protocol for multiple testing, the trial did not meet its primary outcome measure.

**Conclusions and relevance:**

Among patients with atrial fibrillation, heart failure, or treatment-resistant hypertension, the NICC approach was not superior over SoC, despite a significant reduction in all-cause mortality, stroke, myocardial infarction and cardiac decompensation.

*Trial registration* ClinicalTrials.gov Identifier: NCT03317951.

## Introduction

The socio-economic burden of cardiovascular disease (CVD) remains high and even rises for some high-income countries, where it was previously seen to be declining [1]. Therefore, implementing novel interventions is paramount to achieve the 30% reduction in premature mortality due to non-communicable diseases by 2030 (compared to 2015), as defined by the United Nations sustainable development goal (SDG) target 3.4 [2]. The successful achievement of this objective heavily relies on the management of established CVD patients and on those at high-risk for developing CVD. Therefore, early detection and integrated management are paramount to reduce both CVD morbidity and mortality. [3]

Successful patient management is highly dependent on patients’ compliance, which relates to the degree of patients' medication adherence, along with their observance of physician recommendations [4]. The latter is dependent on patients’ perceptions regarding the doctor’s communication with them, as well as doctor–patient relationship [5]. Methods for improving patient compliance consist in a bundle of efforts, such as patient education, self-monitoring, or intensified follow-up using tele-monitoring or remote consultations (e.g., video or telephone calls). For example, heart failure patients can be taught to regularly measure their weight and adjust their therapy accordingly [6]. Atrial fibrillation patients on anticoagulation can self-monitor their INR levels, and arterial hypertension patients can follow-up their blood pressure at home [7]. Recently, the COVID-19 pandemic has forced healthcare providers to move more quickly to e-health consultations, including visits by phone or video. This enabled healthcare providers to address the weaknesses of classic consultations and the realization that numerous chronic disease patients are not well controlled [7].

Tele-monitoring might be one effective approach to reduce CVD burden. Typically, such a tele-monitoring programme comprises patient education, self-monitoring with goal-setting, and regular feedback to improve medical prescription adherence and facilitate lifestyle changes [8]. A recent systematic review, including 26 randomized-controlled trials, showed that tele-monitoring using mobile phone interventions can be a valuable adjunct in CVD care [9].

In Mecklenburg-West Pomerania, a poorly populated federal state in Northern Germany, we developed a novel integrated care concept (NICC) that defines specific patient pathways based on a bundle of interventions for atrial fibrillation (AF), heart failure (HF), and treatment-resistant hypertension (TRH). NICC’s key components are the combination of tele-monitoring and intensive patient care through a call centre. The randomized-controlled CardioCare MV trial, as reported herein, sought to demonstrate NICC’s superiority over standard care (SoC) in patients suffering from either AF, HF, or TRH.

## Methods

### Study design and participants

CardioCare MV was a prospective, randomized, controlled, parallel-group, open-label, blinded-observer trial that aimed to demonstrate NICC’s superiority over SoC according to three hypotheses: (1) NICC is superior to SoC in terms of the combined endpoint comprising mortality, stroke, or myocardial infarction, which should be decreased under NICC versus SoC at 12 month follow-up; (2) NICC results in a lower number of inpatient treatment days versus SoC at 12 month follow-up; (3) NICC results in a lower proportion of patients achieving the combined endpoint of mortality, stroke, myocardial infarction, or cardiac decompensation versus SoC at 12 month follow-up. Details on trial design and statistical analysis plan were formerly reported [10, 11].

Initially, recruited patients were referred by a cardiologist/physician to the recruiting University Medical Centre Rostock (UMR) centre, as were UMR inpatients. Patients aged ≥ 18 years with HF New-York Heart Association (NYHA) II-IV, AF European Heart Rhythm Association (EHRA) II-IV, or TRH with blood pressure ≥ 140/90 mmHg or ≥ 4 anti-hypertensives including at least one diuretic were invited to provide their informed written consent. They were residents of Mecklenburg West Pomerania and members of the health insurance companies *Allgemeine Ortskrankenkasse Nordost* (AOK) or *Techniker Krankenkasse (*NK). NICC patients were to sign an integrated care contract with their health insurance company before study entry. Pregnant or lactating patients and those with cognitive deficits or severe chronic kidney disease were excluded.

The study was conducted according to the most recent Helsinki Declaration version. The protocol was approved by the Ethics Committee of Rostock University Medical Faculty on 18th July 2017 (file number: A 2017–0117); it was registered with drks.de (registration number: DRKS00013124) on 5th October 2017, and secondarily with ClinicalTrials.gov (registration number: NCT03317951) on 17th October 2017. Recruitment started on 1st December 2017.

The project was supervised by both a steering Committee and independent data monitoring committee, which oversaw the safety of care concepts, along with data collection and study conduct.

### Randomisation and masking

The randomisation procedure was described in detail elsewhere [10]. In brief, randomisation was performed according to a 1:1 ratio to NICC or SoC using stratified permuted block randomisation with variable block lengths of 4 and 6. Stratification variables were diagnosed consisting of AF, HF, or TRH, in addition to centre comprising inpatients/outpatients. After checking inclusion/exclusion criteria and signed informed consent forms, the randomisation result was obtained from the trial database so that randomisation was concealed.

### Procedures

NICC: The care centre, available 24/7, was the core of the NICC structure with a central platform for information sharing, care coordination, and patient monitoring. This platform enabled patients, on one side, to provide information from home and care providers, on the other side, to regularly assess the patient’s situation, reviewing their therapy and making adjustments, as necessary, according to the guidelines of the European Society of Cardiology (ESC) [12–14]. Hence, patients dispatched their daily health status via vital sign measurements using a secure communication platform. These incoming data were automatically analyzed using a triage dashboard, with results presented as three-color flags, reflecting the patient’s health status’ urgency.

Before study started, the communication between primary care physicians, medical specialists like cardiologists, and the centre was precisely defined using workflows and care pathways, the main communication tools being telephone calls, faxes, and secured messaging via the platform, depending on respective preferences. Treatment based on NICC ended at 12 months post-randomisation, which was followed by long-term follow-up over another 4 years.

SoC: These patients were treated and followed through conventional consultations with adherence to the guidelines of the European Society of Cardiology (ESC) [12–14].

### Outcome measures

Three primary endpoints were established and measured within the 12 month follow-up period. The first primary endpoint was the composite endpoint of mortality, stroke, and myocardial infarction, the second was the number of inpatient days, the third was the composite endpoint of mortality, stroke, myocardial infarction, and cardiac decompensation.

Secondary endpoints using patient-reported outcomes based on standardized German-translated and validated questionnaires will be reported separately.

### Protocol amendment

The CardioCare MV study protocol was published in 2018 [10]. Because of slow recruitment, the responsible parties endorsed the following changes: recruitment prolongation until 15th August 2019; inclusion of a further 26 cardiologists/internists as part of the UMR study centre; changes in statistical testing procedures. This amended statistical analysis plan (SAP) was presented to the Data Monitoring Committee (DMC), which approved the changes, as did the Ethics Committee of the Rostock University Medical Faculty, and subsequently published [11].

### Statistical analysis

The amended SAP was previously published [11]. Following this amended plan and assuming an annual hospitalization of 0.2 in the NICC group and 0.3 in the SoC group, with an 8.5% drop-out rate, we calculated that 890 patients, corresponding to 445 patients per group, would be required at an 81.8% power to detect any significant effect at a two-sided 5% test level.

Analysis populations for the primary endpoints were the full analysis set (FAS) based on the intention-to-treat (ITT) principle. The full analysis set (FAS) consisted of all randomized patients with informed consent who began their assigned care. Baseline characteristics were summarized as number of patients, with percentages in parenthesis, for categorical variables and as mean, with standard deviation in parenthesis, for continuous variables.

The following hierarchical test procedure was planned. First, the primary endpoint concerning the number of days spent in hospital (hypothesis 2) was to be tested. If this analysis was significant at a two-sided 5% test-level, the full significance level was to fall to the first combined endpoint consisting of myocardial infarction, stroke, or mortality. If no statistical difference was found in the length of hospitalization, then none of the predefined outcome measures were considered significant and superiority of NICC over SoC was not shown. If these two primary endpoints were significant, the full significance was to fall to the last primary endpoint, meaning the combined endpoint of mortality, stroke, myocardial infarction, and cardiac decompensation (hypotheses 3).

Regression analyses were conducted after adjusting for primary disease (AF, HF, and TRH) and centre. The category with the largest numbers was employed as a reference category. Tests were based on the two-sided asymptotic Wald test, with the corresponding 95% confidence intervals (95%CI). Quasi-Poisson regression was applied for count data, logistic regression for the composite endpoints, along with mortality and major adverse cardiovascular events (MACE), defined as myocardial infarction, stroke or cardiovascular mortality. Rate ratios (RR) or odds ratios (OR) were estimated. Secondary analysis included survival analysis for the combined endpoints, mortality, and MACE, where Cox regression was used after adjusting for stratification variables. Kaplan–Meier curves were estimated, and the exact log rank test was calculated.

Based on the amended SAP, core variables for multiple imputations were sex, age, primary disease, centre, and EQ-5D-5L index at baseline. Hospitalization endpoints relied on health insurance data. These data were complete, excepting 38 patients (NICC: *n* = 16, SoC: *n* = 22) who were discarded from analysis. All analyses were conducted using R 4.0.2 in conjunction with R Markdown.

## Results

### Baseline characteristics

**Fig. 1:**
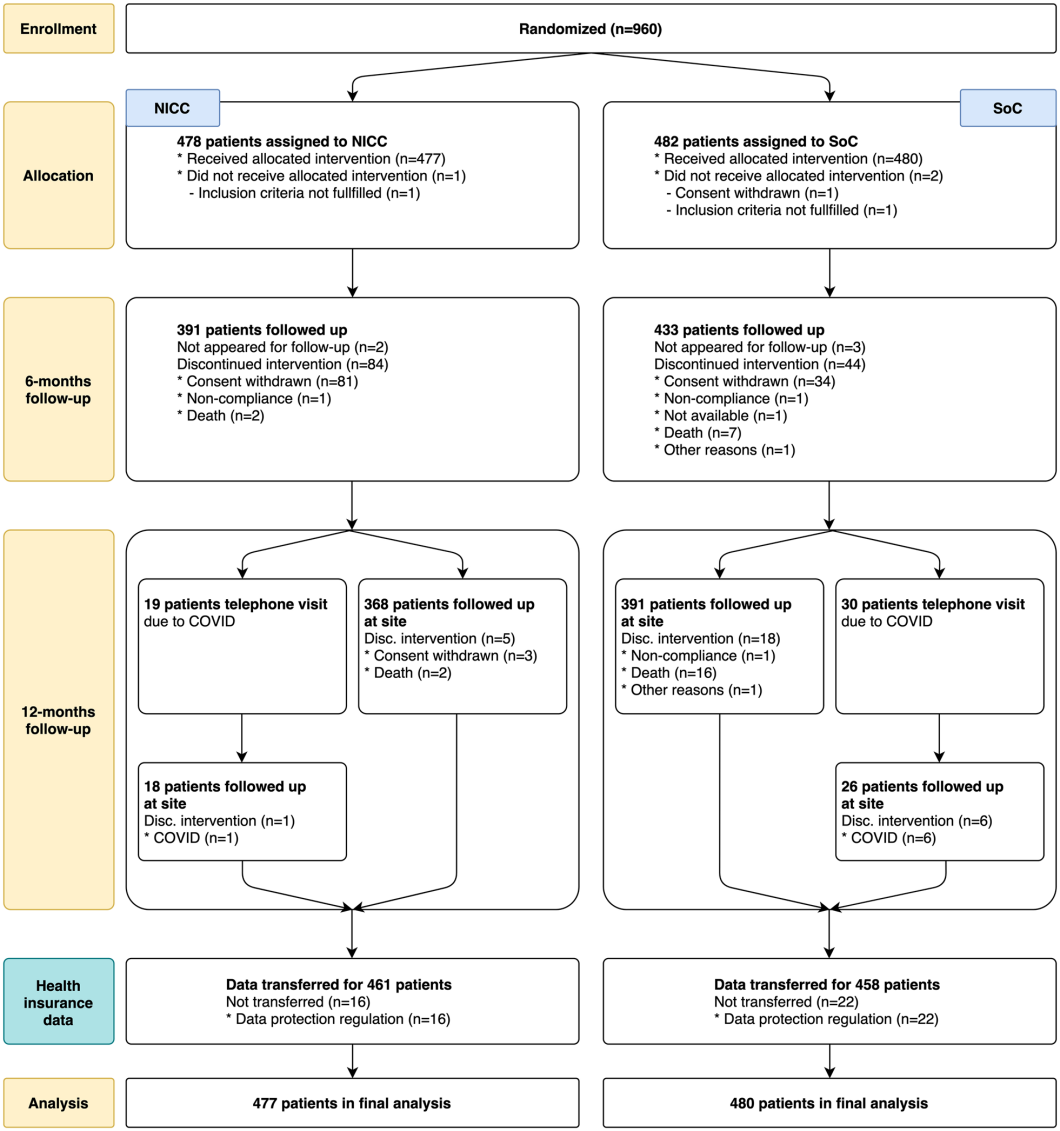
Study flowchart NICC = novel integrated care concept. SoC = standard of care

Between 1st December 2017 and 15th August 2019, 960 patients were randomized (Fig. 1). Follow-up ended as intended approximately one year after the last included patient. Two patients were excluded, as they did not meet the inclusion criteria, and one patient withdrew consent to trial participation. Of the remaining 957 patients, 477 and 480 were assigned to the NICC and SoC groups, respectively. Mean age at randomization was 71 years in both groups, with 59% and 63% being male in the NICC and SoC groups, respectively. Further baseline characteristics are shown in Table 1. The distribution of CVD characteristics among the groups is provided in Table 2.

**Table 1:** Baseline characteristics for the novel integrated care concept (NICC) and standard of care (SoC) treatment groups

	NICC	SoC
Primary diagnosis, prior to randomisation		
Atrial fibrillation	132 (27.7)	133 (27.7)
Heart failure	202 (42.4)	204 (42.5)
Treatment-resistant hypertension	143 (30.0)	143 (29.8)
Inpatient	16 (3.4)	16 (3.3)
Male sex	281 (58.9)	304 (63.3)
Age	71.3 (± 10.4)	71.1 (± 10.8)
Smoking		
Current	45 (9.4)	51 (10.6)
Former	207 (43.4)	232 (48.4)
Never	225 (47.2)	196 (40.9)
Diabetes mellitus	148 (31.0)	158 (33.0)
Physical examination		
Body mass index (kg/m^2^)	29.7 (5.3)	29.9 (5.9)
Systolic blood pressure	137.3 (19.9)	136.8 (20.4)
Diastolic blood pressure	80.0 (10.0)	78.9 (10.8)
Electrocardiogram (ECG)		
Heart rhythm		
Sinus rhythm	347 (72.7)	343 (71.6)
Atrial fibrillation	94 (19.7)	88 (18.4)
Pacemaker ECG	34 (7.1)	43 (9.0)
Other	2 (0.4)	5 (1.0)
Atrioventricular block	65 (13.6)	69 (14.4)
Left thigh block	36 (7.6)	44 (9.2)
Right thigh block	52 (10.9)	47 (9.8)
ECG 2D		
Left ventricular end diastolic pressure (mm)	50.1 (8.3)	49.9 (9.7)
Left ventricular ejection fraction (%)	56.2 (10.9)	55.9 (10.6)
Diastolic dysfunction grade 0 (normal)	145 (37.9)	136 (35.9)
I (impaired relaxation)	172 (44.9)	175 (46.2)
II (pseudo-normal)	42 (11.0)	40 (10.6)
III (restrictive)	4 (1.1)	5 (1.4)
Unknown	20 (5.2)	23 (6.1)

*AHA* American heart association, *CCS* canadian cardiovascular society, *ECG* electrocardiogram, *EHRA* European heart rhythm association, *ESC* European society of cardiology, *NYHA* New York heart association

**Table 2:** Cardiovascular disease characteristics of the novel integrated care concept (NICC) and standard of care (SoC) treatment groups

	NICC	SoC
Cardiovascular diseases		
Atrial fibrillation	210 (44.0)	230 (47.9)
EHRA Stadium I	12 (5.7)	11 (4.8)
II	169 (79.6)	192 (83.5)
III	24 (11.4)	21 (9.1)
IV	0 (0.0)	2 (0.9)
ESC class paroxysmal atrial fibrillation	103 (49.0)	125 (54.3)
ESC class persistent atrial fibrillation	46 (21.9)	55 (23.9)
ESC class long-lasting persistent atrial fibrillation	5 (2.4)	2 (0.9)
Previous hospitalisation due to atrial fibrillation	119 (56.7)	127 (55.2)
Angina pectoris	34 (7.1)	31 (6.5)
CCS Grade I	7 (20.6)	3 (9.7)
II	16 (47.1)	23 (74.2)
III	7 (20.6)	2 (6.5)
Unknown	4 (11.8)	3 (9.7)
Hypertension	341 (71.5)	342 (71.2)
Previous hospitalisation due to hypertension	56 (16.5)	68 (19.9)
Heart failure	213 (44.7)	210 (43.8)
NYHA stadium I	8 (3.8)	5 (2.4)
II	145 (68.1)	137 (65.2)
III	56 (26.3)	67 (31.9)
IV	3 (1.4)	1 (0.5)
Unknown	1 (0.5)	0 (0.0)
AHA stadium A	3 (1.4)	3 (1.4)
B	126 (59.2)	113 (53.8)
C	72 (33.8)	78 (37.1)
D	3 (1.4)	1 (0.5)
Unknown	9 (4.2)	15 (7.1)
Previous hospitalisation due to heart failure	111 (52.4)	116 (55.2)
Medical history		
Coronary artery disease	239 (50.1)	244 (50.8)
Previous myocardial infarction	103 (21.6)	118 (24.6)
Modified CHA_2_DS_2_-VASc score	3.8 (1.7)	3.7 (1.6)
Primary heart valve disease	54 (11.3)	61 (12.7)
Cardiomyopathy	25 (5.2)	34 (7.1)
Cardiac decompensation	42 (8.8)	59 (12.3)
Coronary revascularization	164 (34.6)	162 (33.8)
Bypass surgery	60 (12.6)	71 (14.8)
Heart valve surgery	48 (10.1)	43 (9.0)
Catheter ablation	42 (8.8)	48 (10.0)
Pacemaker	50 (10.5)	50 (10.4)
Defibrillator	27 (5.7)	35 (7.3)
Peripheral artery disease	35 (7.3)	47 (9.8)
Cardiovascular risk factors		
1st degree relative with myocardial infarction < 50 years	57 (11.9)	49 (10.2)

*CCS* canadian cardiovascular society, *ECG* electrocardiogram, *EHRA* European heart rhythm association, *ESC* European society of cardiology, *NYHA* New York heart association

### Primary outcomes

For the first primary endpoint of death, stroke, and myocardial infarction, 6 (1.5%) events occurred in the NICC group and 23 (5.2%) in the SoC group (OR: 3.3 [95%CI 1.4–8.3], *p* = 0.009; Table 3).

**Table 3:** Primary and key secondary endpoints for the comparison of the novel integrated care concept (NICC) with standard of care (SoC)

	NICC		SoC		RR/OR	*p*-value
	Events	Mean/percentage (95% CI)	Events	Mean/percentage (95% CI)		
Primary endpoints						
Death, stroke, or myocardial infarction	6	1.5 (0.6–3.3)	23	5.2 (3.3–7.7)	3.35 (1.36–8.26)	0.009
Number of inpatient treatment days		5.04 (3.95–6.12)		6.51 (4.94–8.09)	1.30 (0.93–1.81)	0.122
Death, stroke, myocardial infarction, or cardiac decompensation	14	3.6 (2.0–5.9)	35	8.1 (5.7–11.1)	2.22 (1.17–4.24)	0.016
Secondary endpoints						
All-cause mortality	4	1.0 (0.3–2.6)	23	5.3 (3.4–7.8)	4.43 (1.55–12.62)	0.006
Time-to-event analysis					5.15 (1.78–14.89)	0.002
Atrial fibrillation	0	0.0 (0.0–3.2)	8	6.7 (2.9–12.7)		
Heart failure	4	2.5 (0.7–6.4)	13	7.2 (3.9–12.0)		
Treatment-resistant hypertension	0	0.0 (0.0–3.0)	2	1.5 (0.2–5.2)		
Cardiovascular death	3	0.8 (0.2–2.2)	15	3.4 (1.9–5.6)	3.85 (1.16––12.81)	0.029
Atrial fibrillation	0	0.0 (0.0–3.2)	5	4.2 (1.4–9.5)		
Heart failure	3	1.9 (0.4–5.5)	9	5.0 (2.3–9.3)		
Treatment-resistant hypertension	0	0.0 (0.0–3.0)	1	0.7 (0.0–4.1)		
Cardiac decompensation	8	2.0 (0.9–4.0)	13	3.0 (1.6–5.1)	1.41 (0.58–3.46)	0.450
Major adverse cardiovascular event (MACE)	5	1.3 (0.4–3.0)	15	3.5 (2.0–5.7)	2.50 (0.89–7.02)	0.083
Time-to-event analysis					3.41 (1.39–8.37)	0.008
Number of hospitalisations		0.71 (0.60–0.83)		0.75 (0.63–0.87)	1.06 (0.85–1.33)	0.603
After adjusting for number of hospitalizations over the 1 year preceding randomisation					1.01 (0.81–1.26)	0.908

Displayed are number of events per treatment group, means or percentages (95% confidence intervals [95% CI] in parenthesis), rate ratio (RR) or odds ratio (OR), 95%CI in parenthesis and *p*-values. Descriptive statistics (n, mean, percentages, corresponding 95% CIs) were obtained from crude complete case analysis*, i.e.,* without adjustments. Rate ratios (RR) and odds ratios (OR) were estimated using quasi-Poisson or logistic regression models after multiple imputation after adjusting for stratification variables

For the number of inpatient treatment days, it was 5.0 (95%CI 4.0–6.1) in the NICC group and 6.5 in the SoC group (95%CI 4.9–8.1) but the difference between the groups did not reach statistical difference (*p* = 0.122).

Finally, the third primary endpoint combining death, stroke, myocardial infarction, and cardiac decompensation showed a difference between the groups, with 14 (3.6%) events recorded in the NICC versus 35 (8.1%) in the SoC group, respectively (OR: 2.2 [95%CI 1.2–4.2], *p* = 0.016). Four patients died from any-cause in the NICC versus 23 in the SoC groups (OR: 4.4 [95%CI 1.6–12.6], *p* = 0.006). Three cardiovascular deaths were recorded in the NICC versus 15 in the SoC groups, respectively (OR 3.9 [95%CI 1.2–12.8], *p* = 0.029). MACEs occurred in 5 NICC and 15 SoC patients, respectively (OR 2.5 [0.9–7.0], *p *= 0.083).

**Fig. 2:**
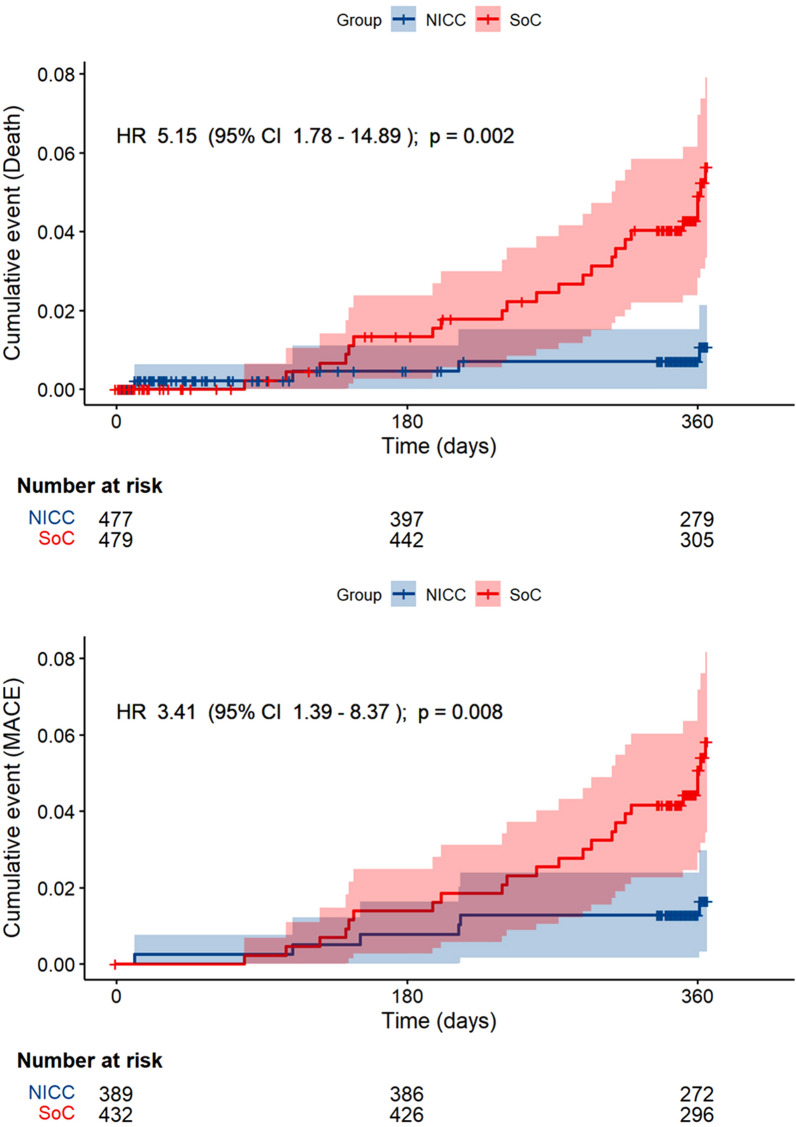
Kaplan–Meier cumulative event curve for all-cause mortality (upper part) and major adverse cardiac events (MACE) (lower part) NICC = novel integrated care concept. SoC = standard of care

Figure 2 depicts Kaplan–Meier curves for all-cause mortality (upper panel) and MACE (lower panel). The between-group differences in survival analysis were significant for all-cause mortality and MACE.

## Discussion

The management of patients with AF, HF or TRH by NICC combining telemedicine with intensive round-the-clock support by a core care centre was not superior over the management of patients using SoC. The lack of benefit was mainly driven by the fact that the number of inpatient days did not differ between the groups. Despite a significant reduction in combined endpoints of (cardiovascular) mortality, stroke, myocardial infarction and cardiac decompensation, the predefined statistical analysis plan dictated a significant test for the number of inpatient days as a prerequisite for superiority of NICC over SoC.

Unlike other studies using controlled designs, we did not record the reasons for hospitalisation, and thus, hospitalization may have been caused by acute events unrelated to cardiovascular conditions (e.g., fractures). Another perspective is that the integrated care concept results in intensified follow-up and may have resulted in early hospitalization in case of slightest doubt about patient safety and well-being. In fact, this preventive and cautious management of patients may have been the sole reason for the highly significant reduction in clinical endpoints without a reduction in the number of inpatients days. Others have also reported similar results for patients with AF using an integrated care concept compared to usual care [15]. In a prospective controlled trial involving frail elderly patients undergoing integrated care at home by a multidisciplinary geriatric team versus usual care, the number of inpatient days did not differ between both groups, while a significant drop was observed in unnecessary hospitalisations, lower cumulative incidence for first emergency room visits, and lower cumulative incidence for first hospitalisations after the first follow-up year [16]. Several trials in HF patients using tele-monitoring programs (versus standard of care) did not show a reduction in HF readmissions or mortality, corroborating our findings [17, 18]. It is very well possible that HF, unlike other chronic diseases, is less suitable for tele-monitoring programs to improve clinical outcome. The feasibility and effectiveness of telemonitoring programs for different chronic diseases requires further investigation.

## Limitations

Our study has several limitations. First, the short observation period caused by funding issues. Long-term clinical outcome and hospitalization rates is key for the assessment of intervention efficacy in chronic diseases. The non-significant difference in inpatient days may have been related to the relatively short follow-up period of 12 months. The planned extended long-term follow-up will provide valuable information. Second, the trial was a single-center experience. For this reason, the data cannot be extrapolated to other (non-tertiary) centers or settings. However, most patients were recruited from the outpatient clinics by physicians involved in cardiovascular care, and thus the study does represent the typical outpatient CVD population.

## Conclusions

Among patients with atrial fibrillation, heart failure or treatment-resistant hypertension, an integrated care concept with tele-monitoring is not superior over standard of care in reducing hospitalization and improving clinical outcome.

## Data Availability

Individual participant data will not be available to third parties because of the data protection contract for the trial. Study protocol and statistical analysis plan have been made available in Trials (2018) 19:120 and Trials (2020) 21:131, respectively.
